# Systematic benchmarking of omics computational tools

**DOI:** 10.1038/s41467-019-09406-4

**Published:** 2019-03-27

**Authors:** Serghei Mangul, Lana S. Martin, Brian L. Hill, Angela Ka-Mei Lam, Margaret G. Distler, Alex Zelikovsky, Eleazar Eskin, Jonathan Flint

**Affiliations:** 1 Department of Computer Science, University of California Los Angeles, 580 Portola Plaza, Los Angeles, CA 90095 USA; 2 Institute for Quantitative and Computational Biosciences, University of California Los Angeles, 611 Charles E Young Drive East, Los Angeles, CA 90095 USA; 3 Department of Psychiatry and Biobehavioral Sciences, David Geffen School of Medicine, University of California Los Angeles, Los Angeles, CA 90095 USA; 4 Department of Computer Science, Georgia State University, Atlanta, GA 30303 USA; 5 The Laboratory of Bioinformatics, I.M. Sechenov First Moscow State Medical University, Moscow, 119991 Russia; 6 Department of Human Genetics, University of California Los Angeles, 695 Charles E. Young, Los Angeles, CA USA

## Abstract

Computational omics methods packaged as software have become essential to modern biological research. The increasing dependence of scientists on these powerful software tools creates a need for systematic assessment of these methods, known as benchmarking. Adopting a standardized benchmarking practice could help researchers who use omics data to better leverage recent technological innovations. Our review summarizes benchmarking practices from 25 recent studies and discusses the challenges, advantages, and limitations of benchmarking across various domains of biology. We also propose principles that can make computational biology benchmarking studies more sustainable and reproducible, ultimately increasing the transparency of biomedical data and results.

## Abstract (web summary)

Benchmarking studies are important for comprehensively understanding and evaluating different computational omics methods. Here, the authors review practices from 25 recent studies and propose principles to improve the quality of benchmarking studies.

## Introduction

Many new algorithms^1,2^ have been developed to accommodate today’s flood of genomic data; however, systematic assessment of software tool performance remains a challenging and laborious process^3^. Without a standardized comparison, potential software users lack an adequate guide for selecting tools that best suit their data. A researcher with a limited computational background may lack sufficient contextual knowledge to weigh the advantages of adopting a new tool, which promises specific gains, against discarding an existing tool with proven capability. Unsystematic assessment of new algorithms creates a communication gap between tool developers and biomedical researchers, the end users of the developed tool.

The developer–researcher gap can be addressed with benchmarking studies, which inform the research community about the most appropriate tools for specific analytical tasks and data types^4,5^. The general purpose of benchmarking is to develop scientifically rigorous knowledge of an analytical tool’s performance^6^, which can be used to guide researchers in selecting a software tool, matching methods with hypothesis formation and testing, and developing tool optimization (i.e., monitor performance as a process control measure).

Assessment of a newly published algorithm is typically performed by the researchers who develop the tool. An unsystematic assessment practice can lead to biases in published results, a phenomenon referred to as the self-assessment trap. Many computational laboratories use simulated data, as they lack adequate resources to generate or access gold standard experimental data when self-assessing a newly developed tool. Using solely simulated data to estimate the performance of a tool is common practice yet poses several limitations. First, the models under which the simulated data are generated can differentially bias the outcomes of algorithms. For example, the algorithm itself could be trained on simulated data prior to running. Second, simulated data cannot capture true experimental variability and will always be less complex than real data^7^. Third, not all simulated data are validated with real-world data, and many methods used to simulate data have yet to be validated by the research community^8^. Even small errors resulting from improperly selected or inaccurately used software tools—or from ignoring the assumptions used by certain tools—can have profound consequences in downstream analyses, potentially producing both false positive and false negative results. A more-comprehensive approach is to complement the simulated data with experimental data, which was generated by the previous studies and is publicly available.

**Fig. 1:**
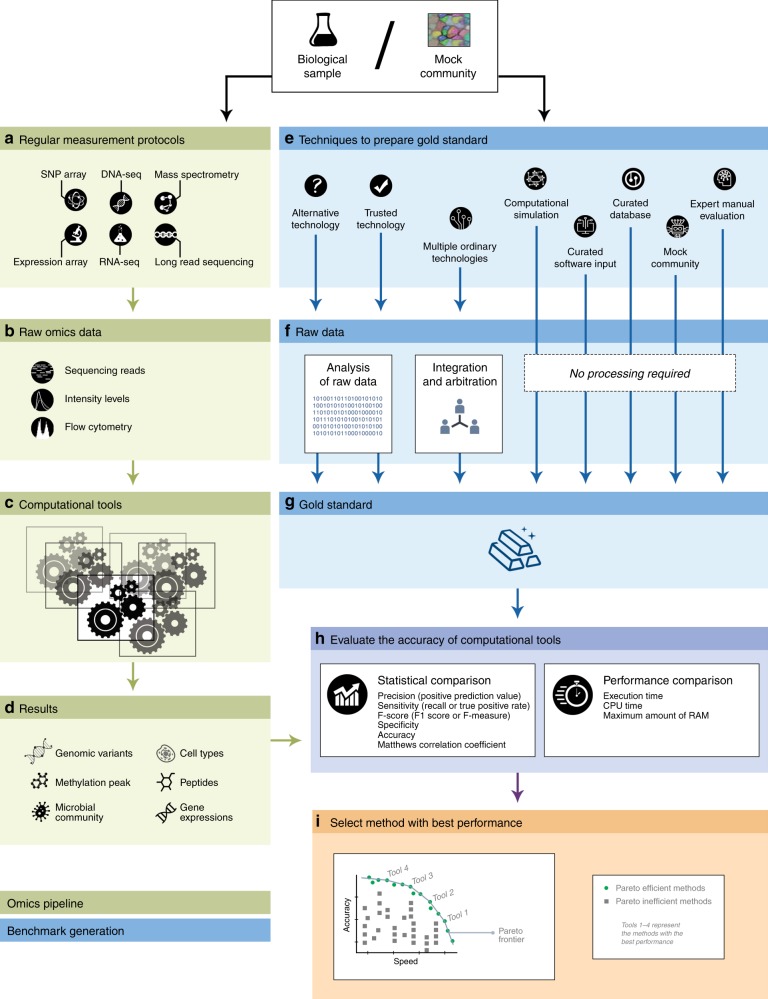
Study design for benchmarking omics computational tools. to evaluate the accuracy of benchmarked computational tools, results obtained by running the computational tools are compared against the gold standard data (ground truth). First, biological samples are probed by regular measurement protocols (processes that generate omics data) (**a**). Raw omics data generated by these protocols serve as the input for examined computational tools (**b**, **c**). Results obtained by running computational tools are the final output of the omics pipeline (**d**). Gold standard data are produced by the benchmarking procedure and are based on technological protocol, expert manual evaluation, synthetic mock community, curated databases, or computational simulation (**e**). (Types of technologies available for use in the preparation of gold standard data are described in the section Preparation of Gold Standard Data.) Some of the techniques used to generate the gold standard data produce raw data, which needs to be analyzed (**f**); other techniques directly produce the gold standard data (**g**). Gold standard data obtained by or in conjunction with the raw omics data generated by regular measurement protocols enables researchers to use statistical metrics (**h**) and performance metrics to assess the computational cost and speed required to run the benchmarked computational tools (**h**), allowing the researcher to draw explicit, standardized comparison of existing computational algorithms. Methods with the best performances are located on the Pareto frontier and are identified as Pareto-efficient methods (**i**). A method is considered to be Pareto efficient if no other benchmarked method improves the score of one evaluation metric without degrading the score of another evaluation metric. (Evaluation methods and criteria for selecting the methods with the best performances are described in the section Selecting a Method with the Best Performance).

Systematic benchmarking based on gold standard data would inform the biomedical research community of the strengths and weaknesses associated with each analytical tool available in computational biology^9^. A benchmarking study first runs available measurement protocols to produce the raw omics data, which serve as the input for the computational tools (see Fig. 1). Results obtained by running computational tools are compared against the gold standard data; comparison of these results with the gold standard allows researchers to use statistical and performance metrics to explicitly differentiate among existing computational algorithms in a standardized way. Ultimately, the generated data and robust scoring methodologies produced by benchmarking studies would be a valuable resource when shared with the research community (Box 1).

Our review summarizes established principles for guiding the design of new benchmarking studies. We separately discuss the challenges and limitations of benchmarking studies and highlight domains of computational biology where, owing to a lack of an accurate gold standard, benchmarking is impossible or limited. We discuss different strategies that can be used to optimize benchmarking, including crowdsourcing and challenge-based benchmarking^10–12^. We also identify and discuss aspects of challenge-based benchmarking relevant to tests performed by individual research groups. Finally, we propose step-by-step instructions for using containerization, common data representation, open data, and systematic parameter description to increase the reusability, transparency, and reproducibility of benchmarking studies.

In this review, we only focus on the benchmarking of the computational tools which inform downstream users about the methods with the best performances. However, benchmarking can be defined more broadly, where estimating the tools with the best performance is only one aspect of the benchmarking. Other goals of the benchmarking may include building community (through competition), stimulating innovation, driving biology, and providing a forum for exchanging ideas and results^13^. Simultaneously balancing all aspects requires concerted effort. For example, a competition-based benchmarking study that publicly releases results on unproven tools could, in some cases, discourage participants from submitted novel tools. In such cases, to keep in mind stimulation of innovation, benchmarking study coordinators may only release prediction results internally. Similarly, in new fields, one cannot always identify proper performance metrics, especially if a challenge is held for the first time.

Box 1 Principles for rigorous, reproducible, transparent, and systematic benchmarking Our review of publications identifies seven principles to guide researchers in designing a benchmarking study that increases reusability, transparency, and reproducibility of benchmarking studies.

1. Compile a comprehensive list of tools to be benchmarked. Identify the software tools that are most suitable for the analytical tasks and data types in the benchmarking project. For example, perform a PubMed (https://www.ncbi.nlm.nih.gov/pubmed/) search for relevant articles. Include software tools mentioned in references of each identified publication. A tool may be impossible to install and run in a reasonable amount of time. We suggest documenting such cases in log files to save the effort of other researchers. (Instructions on how to create log file are provided in Supplementary Note 2.)
  The number of applicable methods may be extremely large; some domains of biology have up to 200 tools currently available^60^. Researchers can scale up a benchmarking study using a competition-based benchmarking model. Alternatively, researchers may review all published tools and select the most popular algorithms for the benchmarking study based on the number of citations or the reputation of the journal. However, selecting the best tool a priori is risky, as tool popularity, journal impact, and citation frequency do not necessarily imply a particular algorithm is optimal^3,61^.
2. Prepare and describe benchmarking data. Maintain a spreadsheet summarizing your benchmarking data. Explain the protocols used for preparing the raw and gold standard data sets. Describe potential limitations of the data. For example, can the benchmarking data bias the performance of any specific type of algorithm? Record methods of benchmarking data preparation, complete provenance, and, if applicable, code for gathering and cleaning data.
3. Select evaluation metrics. Metrics for evaluating the accuracy of software tools need to be carefully selected and packed in the form of scripts, which the community can later use to evaluate the performance of any new algorithms. Benchmarking studies need to consider and document nuances in data representation. For example, comparison of variant calls may be confounded by different representations of insertions, deletions, and multiple nucleotide polymorphisms—particularly when exploring complex regions of the genome^62^.
4. Consider parameter optimization. Parameter optimization is often best understood by the method developers, as deciding how a method is applied to a data set usually involves selecting specific parameters and input pre-processing. In a competition-based model, participants will determine for each tool the optimal parameters. In an independent model, authors need access to all useful combinations of parameters to identify combinations of parameters that perform best for a given algorithm.
5. Summarize algorithm features and share commands for installing and running tools. Update your spreadsheet with the benchmarked algorithm’s features, underlying algorithm, software dependencies, and citation of publication (e.g., Hatem and Ayat et al.^63^). We provide a template of a benchmarking spreadsheet in Supplementary Table 1.
  Provide detailed instructions for installing and running the benchmarked tools. For example, note when a large number of dependencies are required to run a tool. A centralized source of information on issues such as dependencies would be a valuable resource to the research community, as complex computational tasks can be a significant barrier for potential users.
  When many dependencies are required to run a tool, share the benchmarked tool in the form of a computable environment (e.g., virtual machine images, containers, Docker (https://www.docker.com/)^55^). Easy-to-use interfaces that package software with all required dependencies and parameters enable flexibility and portability of the software tool across platforms and operating systems. Consider consulting with tool developers to ensure the correctness of chosen commands, parameters, and other contents in your spreadsheet.
6. Define a universal format (if necessary). When the output of each tool is different, develop and share a script capable of generating a universal format. Data types and formats in many fields of computational biology rapidly change, yet software developers and benchmarking studies can take a lead in standardizing data types and formats. For example, the QfO consortium defines common file formats for orthology inference methods^49^.
7. Provide a flexible interface for downloading data. Sharing an easy-to-use interface that can be used to download the input raw data and the gold standard data helps maximize data reusability. We recommend also sharing the raw output data of each benchmarked tool, so an end user can apply their own evaluation metrics. Scripts available via such interfaces can also be used to reproduce the results and figures of benchmarking studies, ultimately increasing the transparency and computational reproducibility of benchmarking studies^28^.

### Benchmarking studies

A benchmarking study consists of a robust and comprehensive evaluation of the capabilities of existing algorithms to solve a particular computational biology problem. These studies use gold standard data sets to serve as a ground truth and well-defined scoring metrics to assess the performance and accuracy of each tool when applied to a variety of analytical tasks and data types. Gold standard data sets are often obtained using highly accurate experimental procedures that are cost prohibitive in the context of routine biomedical research. For example, Sanger sequencing can be considered a gold standard as it is a highly accurate DNA sequencing technology capable of accurately identifying discrete differences between the human reference and sequencing reads (also known as genetics variants). However, at the time of publication Sanger sequencing costs ~ 250 times more per read than less accurate sequencing platforms.

There is little consensus among researchers about what constitutes a gold standard experimental data set for each particular application (e.g., error correction, genome assembly, microbiome analysis). For example, what is the minimum number of samples that should be included in a benchmarking study? What are adequate levels of coverage and/or fidelity of data? Should there be molecular confirmation of data? These fundamental questions are presently unresolved; systematic benchmarking studies can provide the data and tools to support informed dialog necessary to explore these inquiries.

Owing to the extremely complex nature of biological systems, many domains of modern biology presently lack tools capable of defining and obtaining gold standards. Even when such gold standards are possible to define, producing a gold standard for use in benchmarking studies is an extremely complicated and laborious process^14–16^. (In Supplementary Note 1, we discuss the significant limitations imposed on benchmarking studies by the current lack of an accurate gold standard.) In this section, we summarize three categories of techniques for preparing raw data for the gold standard: techniques involving analysis of raw data, techniques involving an integration and arbitration approach, and techniques that do not require processing of raw data (see Table 1 and Fig. 1e, f).

**Table 1:** Advantages and limitations of various techniques used to prepare gold standard data

Technique	Advantages	Limitations
Trusted technology	High accuracy Direct, usually, no computational inference is required	Carries high cost Does not scale
Alternative technology	Direct, usually, no computational inference is required	Not necessarily more accurate
Multiple ordinary technologies	Using a consensus between the technologies allow reducing the number of false positives compared with each individual technology	Disagreement between used technologies results in the incompleteness of the gold standard
Mock community	Ground truth is fully known, because raw data are generated from prepared gold standard	The small number of items (e.g., microbial species) compared with reality The designed community is artificial
Expert manual evaluation	Most suitable for specialist understanding	Does not scale Lack of formal procedure, limiting comparison of results produced by different experts
Curated database	Allows access to sensitivity, by comparing the number of elements in the sample and the database	Incompleteness of curated databases results in limited ability to define true positives and false negatives
Curated software input	Ground truth is fully known, because raw data are generated from prepared gold standard	Does not validate on real inputs, which usually contain errors
Computational simulation	Ground truth is fully known, because raw data are generated from prepared gold standard Cost-free generation of multiple gold standards	Technology is simulated, and cannot capture true experimental variability and will always be less complex than real data Gold standard data are artificial

Developers can prepare a gold standard by analyzing raw data with currently available technologies (Fig. 1e, f). If possible, a trusted technology (e.g., Sanger sequencing) needs to be applied to a data set in order obtain the gold standard benchmark (Table 1). Trusted technology may not be available; in such cases, alternative technology can be applied. An alternative technology likewise requires minimal or no computational inference, allowing the tools to avoid biases introduced during computational processing of data. In many cases, the accuracy of alternative technologies may be inadequate. The produced gold standards should be applied with caution in such cases. For example, qPCR—widely considered the gold standard for gene expression profiling—shows strong deviations of ~ 5–10% across various targets^17^.

Alternatively, developers can prepare the gold standard with an integration and arbitration approach^4^, which integrates results from multiple ordinary experimental procedures and generates a consensus that serves as a gold standard (Fig. 1e, f). For example, the Genome in the Bottle Consortium (GIB) successfully generated a gold standard reference genome that includes a set of single-nucleotide polymorphisms and small indels by integrating and arbitrating across five sequencing technologies, seven read mappers, and three variant callers^4^. This approach, when compared with each individual technology, is capable of reducing the number of false positives, yet disagreement between used technologies can result in an incomplete gold standard. Such incompleteness challenges the assumption that elements from the gold standard completely overlap with the elements from the sample of the study^18^. Inflated true positive, false positive, and false negative estimates can result when we ignore the fact that some variants present in the sample are missing in the GIB gold standard set results.

Developers preparing a gold standard may choose from several approaches that do not require computational processing of data (Fig. 1e, f). Owing to the complexity of biological systems, it is impossible to obtain the ground truth in many applications (e.g., microbiome analysis). In these cases, instead of obtaining the golden standard, one can design a mock community (often referred to as a synthetic mock community) by combining titrated in vitro proportions of community elements. The most popular mock communities are prepared as mixtures of known microbial organisms^19,20^. When microbial organisms are closely related to similar sequences, such as intra-host RNA-virus populations, one should include closely related pairs and challenge computational methods with various frequency profiles^20–23^. Mock community gold standards offer numerous advantages, but they are artificial and typically comprised of a small number of members when compared with real communities; a smaller number of members increases the risk of oversimplifying reality.

In some cases, expert manual evaluation of output of the technology can be used to produce the gold standard. For example, a trained pathologist can manually evaluate a histological image to determine the infiltration levels of lymphocytes in a tumor. This process allows the pathologist to assign a tumor-infiltrating lymphocyte score—a robust evaluation metric generally supported in the scientific community^24^. Unfortunately, the procedure for manually produced output cannot scale across multiple samples and lacks formal procedure, thereby limiting comparison of results produced by different experts.

Curated databases promise to deliver a highly accurate set of genes, gene variants, and other genomic elements in the form of an encyclopedia. Building large curated databases is a tremendous effort exercised across multiple institutions and agencies and uses a combination of computational analysis, manual annotation, and experimental validation techniques. For example, GENCODE is a database of highly accurate gene features that appear across the entire human genome^25^. Another example of a large curated database is UniProt-GOA, which uses concepts in gene ontology to describe the functions of specific genes^26,27^. Ideally, the content of different gene ontology databases would be synchronized, but, in practice, they have historically contained different annotations^28^.

Such databases can serve as a gold standard, assuming that elements from the database cover the elements from the sample in the study. However, this assumption can be invalid owing to the incompleteness of some large curated databases; the fact that some elements present in the sample are missing in the database may limit our ability to define true positives, false positives, and false negatives. Despite those limitations, large curated databases grant a high level of sensitivity to the researcher by allowing comparison of the number of elements in a sample and in a database.

The challenge of unsystematic benchmarking may, at times, represent a more fundamental problem couched within a multi-step pipeline intended to solve a complex biological problem. The preceding step in a pipeline may introduce errors in its output, of which the succeeding step may not be aware. Then benchmarking of the succeeding step may require as the gold standard a curated software input, in which errors introduced by the previous step are eliminated. For example, the scaffolding problem is a part of the assembly pipeline that starts with assembly of reads into contigs and ends with the output of a scaffold, a set of chains each consisting of ordered oriented contigs bearing estimated gaps between neighbors. The input contigs may be misassembled or may repeat each other; therefore, a “real” benchmark requires curation in order to produce a valid ground truth^29,30^.

At last, researchers can use computational simulation to generate the golden standard, data often referred to as simulated or in silico gold standard data. Simulated data can be generated at no cost, but the application of such data in benchmarking can only complement the real experimental gold standard data. Simulated data cannot be used as a gold standard because it will always be less complex than real data and fails to capture true experimental variability. Lack of an experimental gold standard for the problem of evolutionary inference models and methods (e.g., sequence alignment, phylogenetic tree inference, orthology calling) has resulted in diametrically opposite conclusions provided by different benchmarking studies^30,31^.

Methods designed to simulate experimental data are constantly in development^8^, and numerous attempts have been made to improve the quality of simulated data (e.g., incorporating real and simulated data in one comprehensive, semi-real data set). Analogously, Ewing et al. ^14^ proposed improving the quality of the sequencing data by introducing simulated cancer mutations into real sequencing data. This approach uses real, rather than simulated, sequencing data and maintains the true properties of sequencing reads. Other techniques create semi-real data by subsampling real data sets to generate new data sets with known properties. For example, Soneson et al.^32^ created a null (i.e., no differential expression expected) data set by subsampling from a single group.

Once a gold standard has been prepared for a particular application, the performance of a method can be evaluated using numerous factors (Fig. 1h). Selecting evaluation criteria requires an understanding of statistical assumptions, the differences between an estimate and true performance, the incompleteness of gold standard data sets, and the nature of biased gold standards. This topic is reviewed elsewhere^33^. In this section, we summarize the most commonly used measures that can be used to identify the best-performing methods for a particular analytical task.

Defining statistical measures is a complicated, ambiguous, and context-specific process that requires careful examination. For example, there are numerous ways to define correct alignment of a read against the reference genome. Researchers must decide if the experiment requires that (a) only the start position of the read needs to be correctly mapped, or (b) all bases of the read need to be correctly mapped. More challenging scenarios arise in cases of gapped alignment of RNA-Seq reads, and with the presence of insertion or deletion of bases in the sequence of reads.

According to the confusion matrix, all predictions can be classified as true positives, false positives (i.e., type I error), false negatives (i.e., type II error), and true negatives. These output are the number of correct predictions (i.e., hits), false predictions, missed predictions, and correct rejections, respectively. Once the element from the confusion matrix is defined, one can condense them into various statistical measures. One common measure is precision (i.e., positive predictive value), the percentage of positive predictions made by a test that are true. The other most commonly used measure is sensitivity (i.e., true positive rate or recall), the percentage of known positives that are correctly predicted by a test.

If true negatives are defined, one can calculate the specificity (i.e., true negative rate), accuracy, and Matthews correlation coefficient^34^. Precision and sensitivity are often combined into an F-score (also known as F1 Score or F-measure) measure, a harmonic mean of precision and recall rates. A high F-score indicates a reliably precise and sensitive method. Frequently, the positive or negative prediction is based on a threshold value of a certain parameter which is not always clear how to determine. Rather than assessing prediction just for a single threshold, the performance over a range of cutoffs, including area under the ROC curve or area under the precision-recall curve metrics, is commonly reported.

When the benchmarked method predicts the relative frequencies of members or elements (e.g., microbial species), one can use the correlation between true and predicted relative frequencies to assess each tool’s performance. When few elements are accounting for the majority of frequencies, correlation cannot accurately account for rare items. In such cases, correlation is completely dominated by the most commonly occurring frequencies. To avoid this, one can partition the items in several frequency intervals (e.g., four quartiles) and separately report correlation for each interval. An alternative metric treating all frequencies equally can be represented by the median percent error, which is the computed median of absolute percent errors by which predicted frequencies differ from the true frequencies^35,36^. In some benchmark studies, a binary classification may be insufficient for capturing complexity. For example, a study design may need to predict a structured object (e.g., a consistent subgraph of the gene ontology graph, which would be protein function).

Even the most-accurate software tool will fail to be maximally used if the required computational infrastructure is unaffordable or the program’s running time is exceptionally long. Computational cost of a tool is an important criterion for which there are several means of evaluation. In computer architecture, execution time or runtime is the amount of time a computer spends completing a task. As execution time may vary across different servers, benchmarking studies should report server specifications and number of processors used. The running time of a software package is usually measured in CPU time (i.e., process time). The maximum amount of RAM required to run a software package is a common proxy indicator of required computational resources. Although not easy-to measure, factors critical for the wide adoption of a tool by the scientific community include ease of installing and ease of performing tasks with the tool, as well as availability of analytical options and developers’ timely response to users’ requests.

A benchmarking study can rarely identify a single winner according to all evaluation metrics. Instead, a valid outcome may include identifying multiple methods with excellent performance under different evaluation criteria^37^. We can identify the top-ranked benchmarked tools from the set of Pareto efficient benchmarked methods, which forms a Pareto frontier (Fig. 1i). A method is considered Pareto efficient if no other benchmarked method improves one of the evaluation metrics without degrading another evaluation metric. A benchmarking study cannot blindly classify all Pareto efficient methods as top-ranked tools—how significant one of the evaluation metrics improvement is and how acceptable another metric degradation is should ultimately be subject to expert examination. For example, a method that performs exceptionally well in speed yet mediocre in accuracy—and a method that performed poorly in speed yet exceptionally well in accuracy—could both be identified as Pareto efficient. In such cases, both methods would be reported as winners, and an informed user must use discretion in determining the best method for a given study (see Fig. 1i).

The comprehension and accuracy of a benchmarking study ultimately depends on the quality of work at each step of the benchmarking pipeline outlined in Fig. 1e. The quality of a benchmarking study typically increases with the number of computational tools assessed and consistency of assessment protocol. Our review of current benchmarking practices reflects the most common approach to benchmarking study coordination: the independent model, where single research groups conduct individual benchmarking studies of relevant computational problems. Here, we focus on the challenges, advantages, and limitations of the competition-based model, a less commonly used approach in which participants compete to solve problems in an organized competition.

Challenge-based competitions aim to solve a fundamental research problem in a short period of time by building a scientific community around the topic. First, organizers provide participants with a training data set to develop novel methodologies. Next, participants apply developed methodologies to real data and submit results to a centralized hub, where the evaluation is performed^38^. The limitations and challenges of competition-based benchmarking have been reviewed elsewhere^10,39–41^.

Challenge-based benchmarking was pioneered by Critical Assessment of protein Structure Prediction^42^, the first community-wide contest held in 1994 to assess protein structure prediction methods. The inaugural challenge-based benchmarking event was followed by Critical Assessment of Massive Data Analysis (http://www.camda.info/), the first community-wide experiment in genomics, transcriptomics, metabolomics, and other omics domains^39,42,43^. Since then, community-driven, challenge-based benchmarking efforts have been recognized as effective tools that are capable of enabling the evaluation of novel or existing computational methods^39^.

### A survey of current benchmarking practices

In order to begin identifying and understanding trends in benchmarking of computational omics tools, we surveyed 25 benchmarking studies published between 2011 and 2017. For each study, we documented the area of application (e.g., error correction, genome assembly, microbiome analysis) and the number of tools included in the study (Table 2). To identify trends in benchmarking study design, we noted use of benchmarking study model (e.g., individual, competition-based), raw omics data type (e.g., real, simulated), and gold standard data preparation method (e.g., alternative technology, mock community) (Table 2). In order to assess the types of documentation provided, we assessed whether the published benchmarking study performed parameter optimization, summarized the algorithms’ features, measured the computational cost of the program, shared commands to install and run benchmarked tools, or shared the benchmarking data generated (Table 3). (See legend of Tables 2 and 3 for a detailed account of each criterion.)

**Table 2:** Summary of benchmarking study design and methods

Benchmarking study	Application	No. of tools	Model of study	Raw input data type	Gold standard data preparation method	Parameter optimization
Yang et al. 2013	Error correction	7	I	R	SIMUL	N
Aghaeepour et al. 2013	Flow cytometry analysis	14	C	R	EXPERT	N
Bradnam et al. 2013	Genome assembly	21	C	R	ALTECH	n/a
Hunt et al. 2014	Genome assembly	10	I	R, S	SOFTWARE	N
Lindgreen et al. 2016	Microbiome analysis	14	I	S	SIMUL	No
McIntyre et al. 2017	Microbiome analysis	11	I	R, S	MOCK	N
Sczyrba et al. 2017	Microbiome analysis	25	C	S	SIMUL	n/a
Altenhoff et al. 2016	Ortholog prediction	15	I	DB	DB	Y
Jiang et al. 2016	Protein function prediction	121	C	R	DB	n/a
Radjvojac et al. 2013	Protein function prediction	54	C	R	DB	n/a
Baruzzo et al. 2017	Read alignment	14	I	S	SIMUL	Y
Earl et al. 2014	Read alignment	12	C	R, S	SIMUL	n/a
Hatem et al. 2013	Read alignment	9	I	R, S	SIMUL	Y
Hayer et al. 2015	RNA-Seq analysis	7	I	R, S	ALTECH	N
Kanitz et al. 2015	RNA-Seq analysis	11	I	R, S	ALTECH	N
Łabaj et al. 2016	RNA-Seq analysis	7	I	R	ALTECH	N
Łabaj et al. 2016	RNA-Seq analysis	4	I	R	DB	N
Li et al. 2014	RNA-Seq analysis	5	I	R	ALTECH	Y
Steijger et al. 2013	RNA-Seq analysis	14	C, I	R	ALTECH	n/a
Su et al. 2014	RNA-Seq analysis	6	I	R	ALTECH	Y
Zhang et al. 2014	RNA-Seq analysis	3	I	R	ALTECH	Y
Thompson et al. 2011	Sequence alignment	8	I	DB	DB	N
Bohnert et al. 2017	Variant analysis	19	I	R, S	I&A	Y
Ewing et al. 2015	Variant analysis	14	C	S	SIMUL	n/a
Pabinger et al. 2014	Variant analysis	32	I	R, S	SIMUL	N

Surveyed benchmarking studies published from 2011 to 2017 are grouped according to their area of application (indicated in column “Application”). We also recorded the number of tools benchmarked by each study (“Number of Tools”). We documented the coordinating model used to conduct the benchmarking study (“Model of Study”), such as those independently performed by a single group (“I”), a competition-based approach (“C”), and a hybrid approach combining elements of “I” and “C” (“C, I”). Types of raw omics data (“Raw Omics Data”) and gold standard data (“Gold Standard Data Preparation Method”) were documented across benchmarking study. When a benchmarking study uses computationally simulated data, we marked the study as “S”; when real raw data were experimentally generated in the wet-lab, we marked the study as “R”. When the study used both simulated and real data, we marked the study as “R, S”. Gold standard data types included data that were computationally simulated (marked as “SIMUL”), manually evaluated by experts (marked as “EXPERT”), prepared by alternative technology (“marked as ALTECH”), prepared as curated software input (marked as “SOFTWARE”), prepared as mock community (marked as “MOCK”), prepared from curated databases (marked as “DB”), and prepared using an integration and arbitration approach (marked as “I&A”). In competition-based benchmarking studies, parameter optimization (“Parameter Optimization”) is performed by each team and is not mandatory (marked here as “n/a”). More details about the characteristics of techniques to prepare gold standard data sets are provided in Table 1

**Table 3:** Summary of information types provided by benchmarking studies

Benchmarking study	Application	Summary provided	Computational costs reported	Supporting documentation	Data provided
Yang et al. 2013	Error correction	Y	ExTIME, RAM	N	P
Aghaeepour et al. 2013	Flow cytometry analysis	Y	None	Y	Y
Bradnam et al. 2013	Genome assembly	Y	None	Y	Y
Hunt et al. 2014	Genome assembly	Y	CPU, RAM	Y	P
Lindgreen et al. 2016	Microbiome analysis	Y	ExTIME	Y	N
McIntyre et al. 2017	Microbiome analysis	Y	ExTIME, RAM	Y	P
Sczyrba et al. 2017	Microbiome analysis	Y	None	Y	Y
Altenhoff et al. 2016	Ortholog prediction	Y	None	N	P
Jiang et al. 2016	Protein function prediction	N	None	N	P
Radjvojac et al. 2013	Protein function prediction	Y	None	N	P
Baruzzo et al. 2017	Read alignment	Y	ExTIME, CPU,RAM	Y	P
Earl et al. 2014	Read alignment	N	None	Y	Y
Hatem et al. 2013	Read alignment	Y	ExTIME, CPU,RAM	Y	Y
Hayer et al. 2015	RNA-Seq analysis	N	None	N	P
Kanitz et al. 2015	RNA-Seq analysis	Y	ExTIME, CPU,RAM	Y	Y
Łabaj et al. 2016	RNA-Seq analysis	Y	None	P	Y
Łabaj et al. 2016	RNA-Seq analysis	Y	None	P	Y
Li et al. 2014	RNA-Seq analysis	Y	None	P	Y
Steijger et al. 2013	RNA-Seq analysis	Y	None	P	P
Su et al. 2014	RNA-Seq analysis	N	None	Y	Y
Zhang et al. 2014	RNA-Seq analysis	Y	None	Y	P
Thompson et al. 2011	Sequence alignment	N	None	N	P
Bohnert et al. 2017	Variant analysis	Y	None	Y	P
Ewing et al. 2015	Variant analysis	N	None	N	P
Pabinger et al. 2014	Variant analysis	Y	None	N	N

Surveyed benchmarking studies published from 2011 to 2017 are grouped according to their area of application (indicated in column “Application”). We documented whether benchmarking studies summarized the benchmarked algorithm’s features (“Summary Provided). We recorded whether commands to install and run benchmarked tools were shared (“Supporting Documentation Provided”). We documented whether the benchmarking data are shared publicly (“Data Provided”). We consider the benchmarking data to be fully shared (“Y”) if the gold standard data, raw omics data, and raw output of each benchmarked tool are shared. When any one or more of those data sets is not shared publicly, we recorded the study as partially (“P”). We recorded the computational resources required to run the benchmarked tools (‘Computational Costs Reported”). When the benchmarking study used none of the statistical measures from the confusion matrix, the study was marked as none (“N”). We recorded three measures of computational costs: Execution time (marked as “ExTIME”), CPU time (marked as “CPU”), and the maximum amount of RAM required to run the tool (marked as “RAM”)

We have observed some differences in benchmarking practices across different domains. For example, in the domain of read alignment, there is no feasible mechanism for obtaining the gold standard experimentally. All read alignment benchmarking studies surveyed in this project used computationally simulated data. Similarly, we observed several other domain-specific trends in specific techniques used to simulate gold standard data. For example, surveyed benchmarking studies in the domain of microbiome analysis exclusively used mock community, and the domain of flow cytometry analysis used only expert manual evaluation. On the other hand, we observed that certain gold standard preparation techniques are widely used across domains: computational simulation and curated databases are two methods that carry no cost and were used in benchmarking studies across four different domains.

### Approaches to coordinating a benchmarking study

Most (68%) benchmarking studies are performed by a single research group (see Table 2). In order to generate data, 17 out of 25 surveyed benchmarking studies used the individual model, whereas seven studies used the competitive model. One study included in our review is driven by a hybrid approach that features both benchmarking types.

### Approaches to selecting tools for a benchmarking study

An overwhelmingly large number of software tools are currently available, and an increasing number of applications are released each month. For example, over 200 computational tools have been developed for variant analysis of next-generation genome sequencing data^33^. Independent benchmarking teams would need to invest substantial effort in systematically assessing the accuracy of the extraordinary volume of new analytical methods. Tools are often excluded from a benchmarking study if they lack comprehensive documentation, require a complicated installation process, or are impossible to install and run in a reasonable amount of time^44^. Other benchmarking studies focus only on well-known or frequently used computational tools^45–47^.

On average, each surveyed benchmarking study evaluated 18.3 tools that had been designed to solve a specific problem in computational biology (see Table 2). Benchmarking studies performed under the independent model evaluated an average of 10.7 computational tools, with the total number of tools surveyed by each study ranging from 3 to 32. Competition-based benchmarking studies evaluated an average of 37.3 computational tools; the number of tools evaluated using the competitive model range from 12 to 121 tools.

### Approaches to preparing gold standard data

The most common method used to prepare gold standard data for a benchmarking study is alternative technology; eight out of 25 surveyed benchmarking studies use various alternative technologies (see Table 2). The second most common gold standard preparation technique is computational simulation, observed in eight studies. As previously mentioned, simulated data are not capable of fully capturing true experimental variability and should only be used to complement real gold standard data. Other techniques for gold standard preparation include expert manual evaluation^21^ and curated databases comprised of available databases and literature references^47–50^.

### Approaches to selecting default parameters versus parameter optimization

The process for evaluating a software tool is complex; a researcher must choose specific parameter settings and input pre-processing techniques. Using parameter optimization in a benchmarking study can substantially improve the accuracy of results compared with using default parameter settings. Parameter optimization is computationally intensive and requires running the same tool multiple times, each with different combinations of parameter settings. For example, tuning parameter settings of RNA-Seq aligners is observed to consistently increase the number of correctly mapped reads by an average of 10% across all 14 state-of-the-art aligners^51^. Forty-four percent of surveyed benchmarking studies performed parameter optimization (see Table 2). The remaining benchmarking studies in our review used tools with default parameter settings.

### Approaches to sharing benchmarking data

All the data generated by a benchmarking study offer substantial value to the software development and research community—yet these data are often not shared in the publication nor supplementary materials. Factors contributing to the current low rate of data and code sharing with newly developed methods include an absence of journal policies, requiring the public sharing of these resources and infrastructural challenges to sharing large data generated by the benchmarking studies^52^. Ideally, a benchmarking study should make publicly available all benchmarking data and code necessary to process data and reproduce results^21^.

Although the vast majority of surveyed benchmarking studies are widely disseminated benchmarking data, only 40% of the surveyed studies completely shared benchmarking data (including the raw output of omics tools) (see Table 3). Most studies adopted the “shared upon request” model, which is a less reliable and less reproducible method of data dissemination as it relies on individual authors’ availability to perpetually share data^52,53^. In some circumstances, not sharing data and not being fully transparent is acceptable. For example, in the case of competition-based benchmarking groups with unpublished methods may request not to share their results until the corresponding method paper is published.

### Approaches to sharing supporting documentation

Maximum computational reproducibility of a benchmarking study is only possible when the commands and parameters required to optimally run and install each tool are made publicly available^54^. Providing supporting documentation helps the scientifically community more easily adopt a tool and is particularly important for benchmarked tools that have complicated installation processes or that require prior installation of dependencies^54^. We note that many peer-reviewed journals known for publishing benchmarking studies do not require the sharing of benchmarking data nor supporting documentation.

Only 52% of surveyed benchmarking studies share supporting documentation helpful for a user when installing and running a benchmarked tool (see Table 3). Sharing a tool’s supporting documentation through an easy-to-use interface, rather than through a paper and/or supplementary materials, both of which make it easier for researchers to adopt the method recommended by the benchmarking study^5,55^.

### Approaches to evaluating computational costs

In our review of publications, the vast majority (72%) of benchmarking studies failed to report computational resources and time required to run the evaluated tools (see Table 3).

### Recommendations for a systematic benchmarking study

The interdisciplinary field of computational biology could leverage a systematic benchmarking practice to rapidly assess, disseminate, and implement the many new tools developed and published each month. The results of our review of benchmarking studies published between 2000 and 2017 provide a foundation for discussing potential paths forward in systematic benchmarking of omic computational tools. In addition, benchmarking studies have the potential to combine the strengths of individual tools for a particular application or from a specific technology into a more accurate consensus tool. For example, Aghaeepour et al.^21^ showed that the accuracy of cell population identifications from flow cytometry data can be improved by combining predictions from individual computational algorithms.

### Avoiding overfitting the gold standard data set

Despite the many advantages of reusing benchmarking data, there is a risk of overfitting the developed software to produce the best results on a particular gold standard data set. This process is known as overfitting and can cause the software to produce unreliable results with future data sets. In effort to avoid overfitting, Kanitz et al.^44^ and Altenhoff et al.^49^ have implemented an online interface that evaluates the results of newly developed algorithms. However, mere access to the algorithm evaluation provides no realistic guarantee against algorithm overfitting.

One potential approach to avoiding overfitting is to split benchmarking data into training data and test data, where training data are publicly available and test data are reserved for evaluating the performance of new algorithms. We can detect algorithm overfitting by checking for identical performances of the new algorithm based on the training data and on the test data. Nevertheless, it is important to continuously extend and update gold standards by incorporating novel benchmarks into training and test data sets. This approach avoids overfitting and is capable of meeting newly demanding modifications in usage and technology.

### Parameter optimization

Parameter optimization presents a challenge to independently performed benchmarking studies. Considering even a small number of parameters can produce an intractable number of potential parameter combinations for each tool under study. Several heuristic devices can be used to narrow the search space. The most common narrowing technique involves prioritizing parameters for optimization.

Baruzzo et al.^51^ recommend that benchmarking studies identify parameters that have the most effect on the quality of a tool’s results, then optimize this effect over several combinations of the parameters. For example, the most influential parameter settings for RNA-Seq alignment tools are the number of allowed differences between the reference and the sequencing read, which the tool can tolerate, and seed length^51^. In competition-based benchmarking studies, parameter optimization is optional; such benchmarking studies rely on the expertize of the tool’s developer to choose optimal parameters.

### Sharing benchmarking data

The primary goal of a benchmarking study is to produce a robust assessment of existing algorithms, yet the data generated by benchmarking studies can also be a valuable resource for the research community^56^. Benchmarking data ultimately include gold standard data (Fig. 1g), raw omics data (Fig. 1b), and data generated by benchmarking tools (Fig. 1d).

Access to data generated by benchmarking tools can easily improve the precision of newly developed tools by comparing a new method to the tools previously indexed in the benchmarking study. Results of the benchmarking study can either be downloaded and the analysis locally run, or researchers can upload their own results and obtain a comparison through an online interface^44,49^. In both cases, benchmarking data allow researchers to easily compare newly developed tools against existing tools without installing and running third-party software—often a complicated, time-consuming process, especially when the software lacks detailed documentation^44^.

A particularly effective interface was implemented by The Critical Assessment of Metagenome Interpretation (CAMI)^5^ and is available via GitHub (https://github.com/dkoslicki/CAMIProfilingAnalysis). This approach permanently archives the repositories that store code and data (e.g., Zenodo: https://zenodo.org/) and prevent stored materials from being changed or removed. To the best of our knowledge, only a single published benchmarking study (CAMI)^5^ wrapped tools as portable containers.

### Incentivizing community adoption

Widespread community adoption of a systematic benchmarking practice remains a challenge. In numerous domains of modern biology, recommendations to create benchmarking studies have yet to be adopted by the community. For example, numerous benchmarking studies^51,57^ have established the best-performing RNA-Seq aligners. However, the recommendations of benchmarking studies would have little impact on which RNA-Seq aligners researchers are choosing. TopHat2 was not on the list of the recommended tools because of its relatively long runtime and comparatively low accuracy. Despite the lack of recommendation, TopHat2 was used in at least 30% of published manuscripts based on RNA-Seq data, pushing the developers of TopHat2 to officially announce the retirement of the tool. When researchers choose a less accurate tool, the decision may translate into billions of dollars lost owing to low productivity and any influence the downstream analyses.

Many aspects of benchmarking are open questions. For example, how can we encourage the research community to work on benchmarking while simultaneously working on regular projects (e.g., develop novel computational tools)? Benchmarking is time-consuming and can divert a researcher’s work hours from core scientific projects. Many researchers are concerned that benchmarking work may foster negatively competitive sentiments in the research community and may, ultimately, impact on their careers by discouraging development of new tools. Even if a centralized international organization could manage benchmarking efforts, the source and duration of funding remains open an open problem.

Finally, although funding agencies are interested in novel computational methods, there is little funding available for the benchmarking efforts. Allocating funding for benchmarking research could attract more researchers willing to conduct benchmarking studies. Nevertheless, several feasible mechanisms can promote the attractiveness of benchmarking to researchers. Scientific journals could allocate a special track in each issue for benchmarking papers. Researchers at universities could recruit undergraduate students to test the installation and performance of benchmarked software tools^58,59^.

### Crowdsourcing benchmarking

Individual benchmarking only evaluates published tools, whereas competition-based studies also include novel methods that have not yet been published. A standardized benchmarking approach could use crowdsourcing motivated by competition to develop and run analytical algorithms on proposed data. A competitive crowdsourcing approach has been successfully applied across various domains of computational biology^10^. However, this approach may fail to account for potentially useful tools whose developers did not participate in the competition. In addition, the crowdsourcing approach requires the organization of an active working group, and may not be suitable for independent groups planning to perform extensive benchmarking studies.

### Continuous benchmarking

Benchmarking studies are ephemeral in nature; results can become obsolete in a short period of time as benchmarked data types and analytical techniques decline in use. The fast pace of new method development and publication dictates the need for continuous benchmarking. Further, benchmarking is only able to evaluate methods implemented in a current release of the software. New releases of a method can potentially differ in accuracy and runtime, suggesting a community-wide need for a permanent benchmarking effort^58^. In addition to accounting for new method development, benchmarking practice also needs to incorporate changes in reference databases (such as Gene Ontology)^48^. Routinely updating a benchmarking study may require that developers determine the intersection between the previous and current versions of the databases. None of the bioinformatics problems should be considered as solved at any given point in time; continuous benchmarking needs to be performed in order to inform the user about the best algorithms currently available for a problem.

## Discussion

Following our proposed practices would help biomedical researchers leverage the current technological expansion to optimize accuracy and potential of their projects. The life science and biomedical research community is interested in systematic benchmarking of previously published methods, but running algorithms developed by other researchers is a challenging task for tools with many dependencies and limited documentation. The extraordinary volume of new analytical methods that are published each month compounds the challenge of accurately testing each tool. These challenges should not discourage the research community from performing systematic benchmarking studies of computational biology methods. Instead, these challenges motivate the need for clearly articulated, transparent, systematic, and standardized benchmarking practices. Proposed principles will make computational biology benchmarking studies more sustainable and reproducible, ultimately increasing the transparency of biomedical data and results.

## Supplementary information

Supplementary Information
