# Supplementary material for: Systematic benchmarking of omics computational tools
Source: Nat Commun. 2019 Mar 27;10:1393. doi: 10.1038/s41467-019-09406-4 (PMC6437167; doi:10.1038/s41467-019-09406-4)
Supplement: Supplementary file 1 — Supplementary Information [file 41467_2019_9406_MOESM1_ESM.pdf]

SUPPLEMENTARY INFORMATION

Supplementary Note 1: Challenges to generate an accurate gold standard

While many areas of biological science, including RNA biology, currently lack a gold standard, some areas of biological science have developed successful benchmarking computational tools that produce an accurate gold standard. Examples of successful benchmarking studies include specific problems in DNA biology, such as variant calling [14,60] or genome assembly [37]. These studies' achievements can inaccurately portray benchmarking as a straightforward problem, where researchers first devote effort to generate the gold standard data sets, and then make reliable decisions based on uniform statistical methods. In contrast to DNA biology, many problems in RNA and protein biology involve extremely complex systems, making the definition and acquisition of gold standards extremely challenging or impossible.

Scientific problems in RNA biology, one example of such a complex system, still center around determining differentially expressed genes from RNA-Seq data. Solving this problem involves multiple steps: (1) alignment of short reads to the reference genome and/or transcriptome; (2) gene and/or isoform quantification; (3) normalization of gene or isoform expression levels; and (4) differential expression analysis. Each step of the analysis has a major impact on the final set of differentially expressed genes and carries unique challenges, which we discuss below. The first step of differential expression analysis is the read alignment problem—to find the correct genomic location of tens of millions of sequencing reads derived from RNA transcripts. We currently lack experimental techniques capable of generating an accurate gold standard of true

read alignments. In fact, RNA biology is an area of study where one can realistically argue that simulated data is the only alternative available when preparing a gold standard [51].

The second step of differential expression analysis is the transcriptome quantification problem—to identify the gene and isoform from which each read was originated, and how to use those reads to quantify the expression levels of genes and RNA isoforms. True expression levels of isoform and genes are impossible to measure even in a simple bacterial organism, where RNA transcripts are not subject to alternative splicing. Human RNA transcripts undergo alternative splicing, which presents an even more substantial challenge to obtaining a gold standard. Lack of a gold standard for gene and isoform expression levels forces the biomedical community to adopt alternative technologies for obtaining a gold standard. Measurements of gene and isoform expression levels obtained by alternative technology should not be considered a true set, as they have their own inherent biases and limitations. For example, qPCR—widely considered the gold standard for gene expression profiling—has been shown to exhibit strong deviations of ~5-10% across various targets [17].

The third step of differential expression analysis is the expression normalization problem—to remove the biases and the variance introduced by experimental issues, while preserving the true biological variation. Currently, we lack experimental techniques capable of estimating true biological variation and differentiating variation from technical noise. Current RNA-seq analysis methods typically standardize data between samples by scaling the number of reads in a given library to a common value across all sequenced libraries, which is an oversimplification for many biological applications [64]. Lack of a gold standard prevents the biomedical research

community from assessing the performance of the tools that measure biological and technical variance [65].

The final step of differential expression analysis is to determine differentially expressed (DE) genes. This problem involves running a large number of hypothesis tests in parallel, one for each gene or isoform. To properly benchmark this problem, one needs to vary multiple parameters, including the number of replicates, the number of DE genes, and the effect sizes. Nonetheless, the accurate gold standard cannot be obtained by current experimental procedures. The complexity of the differential expression analysis problem prevents the level of comprehension needed in a benchmarking study to evaluate all steps of RNA-Seq analysis. Instead, benchmarking studies separately evaluate each step of the problem [45].

Lack of an accurate gold standard imposes a significant limitation on benchmarking studies. Researchers planning to perform the benchmarking study face a dilemma, where, on one hand, they do not have access to experimental techniques to generate accurate gold standard, and, on another hand, it is known that the extreme complexity of the problem cannot be captured by simulated data. One compromise is to enhance the simulated data with the real data or to adjust the real data to the needs of the benchmarking study using computational techniques.

Supplementary Note 2: An example of a log file for a software tool installation and running

The log file includes any necessary dependencies and documents needed for the process of installing the software tools and corresponding dependencies. Include any errors that occurred while installing dependencies and the commands used to overcome these installation problems. The log file documents the type of files that needed in order to input data into the tools and the format of the output file.

This is the possible structure of the log file:

- Input
- Output
- Dependencies
- Commands used to install the tool
- Commands used to run the tool
- Reason the tool is impossible to install. This should include the exact error message and document steps (if any) which were performed to resolve the problem. In case the software developers were contacted, their suggestions should be listed here.

Supplementary Table 1. Summary of error correction algorithm features.

Software tool	Version	Underlying algorithm	Data structure	Types of reads accepted
BLESS	1.02	k -mer spectrum	Bloom filter and hash table	SE/PE
Fiona	0.2.8	k -mer spectrum	Partial suffix array	SE
Pollux	1.0.2	k -mer spectrum	Hash table	SE/PE
BFC	1	k -mer spectrum	Bloom filter and hash table	SE/PE
Lighter	1.1.1	k -mer spectrum	Bloom filter	SE/PE
Musket	1.1	k-mer spectrum	Bloom filter and hash table	SE/PE
Racer	1.0.1	k-mer spectrum	Hash table	SE/PE
Reptile	1.1	k-mer spectrum	Hamming graph	SE
Quake	0.3	k-mer spectrum	Bit array index	SE/PE
SOAPdenovo2 Corrector	2.03	k-mer spectrum	Hash table	SE/PE
ECHO	1.12	Multiple sequence alignment	Hash table	SE/PE
Coral	1.4.1	Multiple sequence alignment	Hash table	SE/PE
RECKONER	0.2.1	k-mer spectrum	Hash table	SE
SGA	0.10.15	FM-index search	FM-index	SE/PE
ShoRAH	1.1.0	clustering	Not specified	SE
KEC	1	k -mer spectrum	Hash table	SE

Supplementary Table 2. Summary of error correction algorithm features and publication details.

**Software tool**	**Organism**	**Journal**	**Publication Year**
BLESS	Human, *E. coli*, *S. aureus*	Bioinformatics	2014
Fiona	Human, *Drosophila* sp., *E. coli*, *C. elegans*	Bioinformatics	2014
Pollux	Human, *E. coli*, *S. aureus*, mixed genome data	BMC Bioinformatics	2015
BFC	Human, *C. elegans*	Bioinformatics	2015
Lighter	Human, *E. coli*, *C. elegans*	Genome Biology	2014
Musket	Human, *E. coli*, *C. elegans*	Bioinformatics	2012
Racer	Human, *Drosophila* sp., *E. coli*, *C. elegans*, other bacteria	Bioinformatics	2013
Reptile	Human, *Acinetobacter* sp., *E. coli*	Bioinformatics	2010
Quake	Human, *E. coli*	Genome Biology	2010
SOAPdenovo2 Corrector	Human, PhiX174, *Drosophila* sp., *Saccharomyces cerevisiae*	Giga Science	2012
ECHO	Human	Genome Research	2012
Coral	Human, *E. coli*, *S. aureus*	Bioinformatics	2011
RECKONER	Human, *S. cerevisiae*, *C. elegans*, *M. acuminata*	Bioinformatics	2017
SGA	Human, *C. elegans*, *E. coli*	Genome Research	2012
ShoRAH	RNA viral population	BMC Bioinformatics	2011
KEC	RNA viral population	BMC Bioinformatics	2012

Supplementary Table 3. Summary of error correction algorithm programming language and comparable software tools.

**Software tool**	**Programming language**	**Programs compared to in the publication**
BLESS	C++	SGA, QuorUM, Lighter, BFC, DecGPU, ECHO, HiTEC, Musket, Quake, Reptile
Fiona	C++	Allpaths-LG,Coral,H-Shrec,ECHO,HiTEC,Quake
Pollux	C	Quake, SGA, BLESS, Musket, RACER
BFC	C	BLESS, Bloocoo, fermi2, Lighter, Musket, and SGA
Lighter	C++	Quake, Musket, Bless, Soapec
Musket	C++	SGA, Quake
Racer	C++	Coral, HITEC, Quake, Reptile, SHREC
Reptile	C++	SHREC
Quake	C++, R	SOAPdenovo,EULER, SHREC
SOAPdenovo2 Corrector	C/C++	SOAPdevnovo1, ALLPATHS-LG
ECHO	Python	SA, SHREC
Coral	C	COMPASS 3.0, HHalign 1.5.1.1 and PSI-BLAST
RECKONER	C++	Ace, BFC, BLESS, Blue, Karect, Lighter, Musket, Pollux, RACER, Trowel
SGA	C++	Velvet, ABySS, SOAPdenovo, Quake, HiTEC
ShoRAH	C++, Python, Perl	No comparison included
KEC	Java	ShoRAH

Supplementary Table 4. Summary of published URLs for each software tool webpage.

**Software tool**	**Tool webpage**
BLESS	https://sourceforge.net/p/bless-ec/wiki/Home/
Fiona	https://github.com/seqan/seqan/tree/master/apps/fiona
Pollux	https://github.com/emarinier/pollux
BFC	https://github.com/lh3/bfc
Lighter	https://github.com/mourisl/Lighter
Musket	http://musket.sourceforge.net/homepage.htm
Racer	http://www.csd.uwo.ca/~ilie/RACER/
Reptile	http://aluru-sun.ece.iastate.edu/doku.php?id=reptile
Quake	http://www.cbcb.umd.edu/software/quake
SOAPdenovo2 Corrector	http://soap.genomics.org.cn/about.html
ECHO	http://uc-echo.sourceforge.net/
Coral	https://www.cs.helsinki.fi/u/lmsalmel/coral/
RECKONER	https://github.com/refresh-bio/RECKONER
SGA	https://github.com/jts/sga
ShoRAH	https://github.com/cbg-ethz/shorah
KEC	http://alan.cs.gsu.edu/NGS/?q=content/pyrosequencing-error-correction-algorithm

Supplementary Table 5. Summary of software dependencies and other features.

Software tool	Software dependencies	Default k-mer size	Read trimming
BLESS	MPICH 3.1.3, OpenMPI 1.8.4, Boost library, google spareshash, klib, KMC, murmurhash3, zlib, pigz	N/A	YES
Fiona	N/A	N/A	YES
Pollux	64 bit Unix-based OS	31	YES
BFC	N/A	N/A	NO
Lighter	N/A	N/A	NO
Musket	N/A	N/A	NO
Racer	OpenMP	N/A	NO
Reptile	Perl, GNU make, C++ compiler	24	NO
Quake	N/A	15	YES
SOAPdenovo2 Corrector	GCC 4.4.5 or later	N/A	N/A
ECHO	GCC 4.1 or later, Python 2.6, numpy, scipy	1/6 of read length	YES
Coral	N/A	N/A	YES
RECKONER	KMC2, KMC tools	N/A	NO
SGA	Google sparse hash library, bamtools, zlib, jemalloc (optional), pysam, ruffus	31	NO
ShoRAH	Biopython, NumPy, Perl, zlib, pkg-config, GNU scientific library	N/A	YES
KEC	FAMS; ClustalW2 or Muscle (optional)	25	NO
